# Bibliographical

**Published:** 1873-01

**Authors:** 


### BIBLIOGRAPHICAL.

*Contagious Diseases.* By JOHN MORGAN, A.M., M.D., Fellow of the Royal College of Surgeons, Ireland, etc. Philadelphia: J. B. Lippincott & Co., Publishers.

Under the above title, we have practical lessons in the nature and treatment of the affections produced by the contagious diseases, with an account of the primary syphilitic poison, and of its communicability—based on extensive, direct and comparative observation of the disease in both sexes; with an appendix on the recent report of the royal commission on the contagious diseases act, and its application to the voluntary hospital system.

The contagious diseases known as venereal have been so much discussed of late, with regard to the repression of the extension of these maladies by compulsory legislation, and more particularly as to the advantages and efficiency of such an infringement on the liberty of even the depraved portion of the people, with the view of restraining by force the propagation of these poisons, that the discussion of this question by so intelligent an observer as the author evidently is, must be interesting to every medical man.

*Angular Curvature of the Spine.* By BENJAMIN LEE, A.M., M.D., of Philadelphia. J. B. Lippincott & Co., Publishers.

The correct treatment for angular curvature of the spine is certainly most important information. This volume proposes to furnish it. The prominent points set forth as established in the author's experience are: that in a great number of cases an early recognition of the disease will enable us to arrest it before deformity has been produced; that mechanical treatment is of vastly greater importance than medication; and that the form of mechanical treatment which gives the most satisfactory results is that of constant anterio-posterior support by portable instruments, assisted by occasional modified suspension by means of fixed apparatus. A table is appended which indicates the diseases with which angular curvature is most frequently confounded in its initial stage.

*Injuries of Nerves, and their Consequences.* By S. WEIR MITCHELL, M.D., Member of the National Academy of Sciences, Physician to the Philadelphia Orthopædic Hospital and Infirmary for Diseases of the Nervous System, etc. Philadelphia: J. B. Lippincott & Co., Publishers.

This book is the result of the experience of the author in a special hospital established during the late civil war by Dr. W. A. Hammond, then Surgeon-General of the United States Army. Among the vast number of cases treated in this hospital, were the rarest forms of nerve lesion of almost every great nerve in the human body, and new modes of treatment were devised and observations made of the results at the time, while, in many instances, the utmost care has been taken to collect, in the interval which has elapsed since the war, such details of later history as were needed to clear up and complete the story of symptoms or prognosis. We can commend the work as one of rare value.

*Obstetric Aphorisms for the use of Students Commencing Midwifery Practice.* By JOSEPH GRIFFITHS SWAYNE, M.D., Physician Accoucheur to the Bristol General Hospital, etc. Second American from the fifth revised English Edition, with Additions by Edward R. Hutchins, M.D. Philadelphia: Henry C. Lea, Publisher.

The object of this work is expressed perhaps sufficiently in its title—viz, to give a few brief and practical instructions to the novice in obstetrics in the conduct of ordinary cases of labor, and also to indicate to him in unusual cases what should be his course. It is not intended to supply the place or supersede the regular works upon obstetrics, but to serve the important purpose of a guide to beginners in the obstetric art, and to guard them against either temerity or timidity. It contains chapters on the management of ordinary labor, cases in which the student ought to send for assistance, diseases of pregnancy, care of the new-born infant, and abortions, in which is embodied much that is valuable to the experienced practitioner.

*Hand-Book of Compound Medicines, or the Prescriber's and Dispenser's Vade-Mecum.* By ARNOLD J. COOLEY. Philadelphia: J. B. Lippincott & Co., Publishers.

This is a book of formulæ from pharmacopœias, hospitals, leading physicians, and formulæ and notices of proprietary preparations and nostrums, which we cannot commend as in any way calculated to advance medical science; for, independent of other serious objections, while professing "to show the actual value of the pretentious nostrums on which many persons so confidently and unwittingly spend their money," it furnishes the facilities for the propagation of this traffic.

